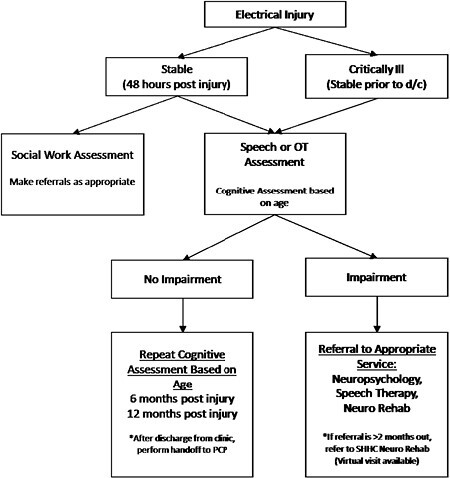# 771 Clinical Practice Guideline: Standard of Care for Cognitive Screening/Evaluation of Patients Following Electrical Injuries

**DOI:** 10.1093/jbcr/irae036.312

**Published:** 2024-04-17

**Authors:** Hadley A Regal, Kristina Frazier, Mindy Orr, Crystal D Webb, Callie M Thompson

**Affiliations:** University of Utah Health, Salt Lake City, UT; University of Utah Health Center, Farmington, UT; University of Utah, Cottonwood Heights, UT; University of Utah Health, West Haven, UT; University of Utah Health, Salt Lake City, UT; University of Utah Health Center, Farmington, UT; University of Utah, Cottonwood Heights, UT; University of Utah Health, West Haven, UT; University of Utah Health, Salt Lake City, UT; University of Utah Health Center, Farmington, UT; University of Utah, Cottonwood Heights, UT; University of Utah Health, West Haven, UT; University of Utah Health, Salt Lake City, UT; University of Utah Health Center, Farmington, UT; University of Utah, Cottonwood Heights, UT; University of Utah Health, West Haven, UT; University of Utah Health, Salt Lake City, UT; University of Utah Health Center, Farmington, UT; University of Utah, Cottonwood Heights, UT; University of Utah Health, West Haven, UT

## Abstract

**Introduction:**

Electrical injuries account for nearly 3% of burn center admissions and most electrical injuries occur in the workplace; 55% according to the latest ABA Burn Injury Summary Report. No guideline(s) currently exist for a standard of care for comprehensive cognitive screening/evaluation and management of patients after electrical injuries. Research indicates that almost 50% of all patients who experience an electrical injury (without need to delineate low vs. high voltage injuries) report physical, cognitive, and/or emotional/psychosocial challenges after their injuries. These reported cognitive impairments most commonly include difficulty within attention (sustained/alternating/divided), memory (immediate, working, and delayed recall), new learning, and word finding abilities. We sought to utilize existing literature to develop a care pathway for our burn center.

**Methods:**

We performed a literature review and found consistent reports of varying cognitive impairments/decline following electrical injury. Given the lack of a standard of care, we utilized this existing literature to formulate the care pathway in Figure 1 for patients (pediatric through geriatric) that were admitted to our burn center.

**Results:**

We presented the proposed workflow in our monthly Burn Administration meeting and all leaders from the interdisciplinary team agreed on the pathway and for its immediate rollout. This pathway includes screening tool options, comprehensive standardized testing for both pediatric populations as well as adult/geriatric individuals. We aimed to have repeatable testing options that were age appropriate and could also be completed in additional languages to support various patient demographics.

**Conclusions:**

All patients who sustain an electrical injury receive an immediate consultation to Speech Language Pathology upon admission to our burn center for comprehensive screening and evaluation of their cognitive/linguistic profile. Careful selection of testing protocols is completed based on age. Upon completion of testing, cognitive retraining is initiated as appropriate while in the ICU setting; as indicated, cognitive retraining continues in the outpatient setting. Patients may also be referred to Neurorehabilitation and Neuropsychology/Neuropsychiatry.

**Applicability of Research to Practice:**

This workflow has both immediate clinical applicability and will allow us to both set expectations with patients about the typical post injury progression of cognitive function and to intervene to achieve optimal neuropsychological outcomes.